# Two-step Mendelian randomization reveals a lipid-driven protective effect of type 2 diabetes on ALS

**DOI:** 10.1007/s10072-025-08407-0

**Published:** 2025-08-18

**Authors:** Mingjun Kong, Weiyi Yu, Jianhui Guo, Zhuoya Wang, Dongsheng Fan

**Affiliations:** 1 Department of Neurology, Peking University Third Hospital, Beijing, China; 2 Beijing Key Laboratory of Biomarker and Translational Research in Neurodegenerative Diseases, Beijing, China; 3 Key Laboratory for Neuroscience, National Health Commission/Ministry of Education, Peking University, Beijing, China; 4 Department of Neurology, Key Laboratory of Neurogenetics and Channelopathies of Guangdong Province and the Ministry of Education of China, Institute of Neuroscience, Guangzhou, Guangdong China; 5 The Second Affiliated Hospital, Guangzhou Medical University, Guangzhou, Guangdong China; 6 Institute of Child and Adolescent Health, School of Public Health, Peking University, Beijing, China; 7 National Health Commission Key Laboratory of Reproductive Health, Beijing, China

**Keywords:** Amyotrophic lateral sclerosis, Type 2 diabetes mellitus, Mendelian randomization, Lipid metabolism, Serum metabolites, Neurodegeneration

## Abstract

**Background:**

Amyotrophic lateral sclerosis (ALS) is a progressive neurodegenerative disorder with few therapeutic options. Observational data suggest that type 2 diabetes mellitus (T2DM) might protect against ALS, yet the mechanisms are unclear. Clarifying whether glucose or lipid metabolism underpins this protective effect could guide targeted interventions.

**Objective:**

This study aims to investigate if T2DM reduces ALS risk through glycemic or lipid pathways using a two-step Mendelian Randomization (MR) approach.

**Methods:**

Summary-level genetic data were sourced from FinnGen (*n* = 440,735), MAGIC (*n* = 200,622), UK Biobank (*n* = 115,078), and Project MinE (*n* = 138,086). Two-sample MR assessed T2DM’s causal effect on ALS, followed by multivariable MR adjusting for glycemic traits to identify metabolic pathways. A two-step MR analyzed significant blood metabolites contributing to the T2DM-ALS relationship. Sensitivity analyses confirmed the robustness of these findings.

**Results:**

T2DM exhibited a protective causal association with ALS (inverse variance weighting OR = 0.956, 95% CI 0.916–0.997, *p* = 0.037). Glycemic traits did not mediate this protection; instead, lipid metabolism played a role. Specifically, a 1 SD reduction in LDL diameter was linked to a 16.7% decrease in ALS risk, accounting for 24.4% of T2DM’s protective effect. Similarly, a 1 SD decrease in total esterified cholesterol (TEC) reduced ALS risk by about 13.2%, contributing to 13.3% of T2DM’s overall protective impact. No evidence of horizontal pleiotropy was observed.

**Conclusion:**

T2DM’s protective influence on ALS primarily involves lipid rather than glucose pathways, highlighting TEC and LDL particle diameter as crucial mediators. Targeting lipid metabolism may offer new therapeutic strategies to reduce ALS risk or progression, potentially leading to focused nutritional interventions and biomarker development.

**Supplementary Information:**

The online version contains supplementary material available at 10.1007/s10072-025-08407-0.

## Introduction

### Overview of ALS and the metabolic paradox of T2DM

Amyotrophic lateral sclerosis (ALS) is a fatal neurodegenerative disorder characterized by progressive degeneration of motor neurons, leading to muscle weakness, atrophy, and significant disability. Its prevalence is rising, partly due to an aging population and improved supportive care. Despite extensive research, current ALS treatments offer limited benefit, highlighting an urgent need for novel therapeutic approaches [1–3]. Intriguingly, emerging epidemiological evidence consistently indicates that type 2 diabetes mellitus (T2DM) is associated with a reduced risk of developing ALS [4–7], suggesting a potential protective effect. However, the biological mechanisms underlying this inverse relationship remain poorly understood.

### Metabolic dysregulation and hypothesized protective mechanisms

Metabolic dysregulation, including weight loss, hypermetabolism, and dyslipidemia, is a feature of ALS linked to disease progression and survival. The inverse association between T2DM and ALS risk underscores the critical role of metabolic pathways in pathogenesis. Two hypotheses may explain this protective effect: first, chronic hyperglycemia might alleviate mitochondrial bioenergetic deficits in motor neurons; second, insulin resistance could induce protective metabolic adaptations, such as enhanced utilization of alternative energy substrates [8]– [9]. The distinct roles of glucose and lipid pathways in ALS pathogenesis have therapeutic relevance. Glucose metabolism deficits primarily impair ATP production and antioxidant capacity, while lipid dysregulation affects membrane integrity and signaling.

### Employing Mendelian Randomization to establish causality

Establishing causal links between T2DM, metabolic factors, and ALS is challenging using observational data alone due to confounding and reverse causality. Mendelian randomization (MR) analysis addresses these limitations by leveraging genetic variants as instrumental variables to infer causality [10]. This approach utilizes genetic data as proxies for exposures, effectively simulating a randomized trial.

### Hypothesis and study objectives

We hypothesize that T2DM confers protection against ALS via alternative metabolic pathways, particularly involving lipid metabolism and fatty acid oxidation, beyond alterations in glucose metabolism. Using MR analysis of large-scale genome-wide association study (GWAS) data, our primary objectives are to: (1) Elucidate the mechanisms linking T2DM and ALS; (2) Identify potential intermediary blood metabolites within this causal pathway; and (3) Explore the therapeutic potential of targeting these metabolite pathways. This research aims to provide robust evidence to inform the development of novel metabolic strategies for ALS.

## Materials and method

### Study design

**Fig. 1:**
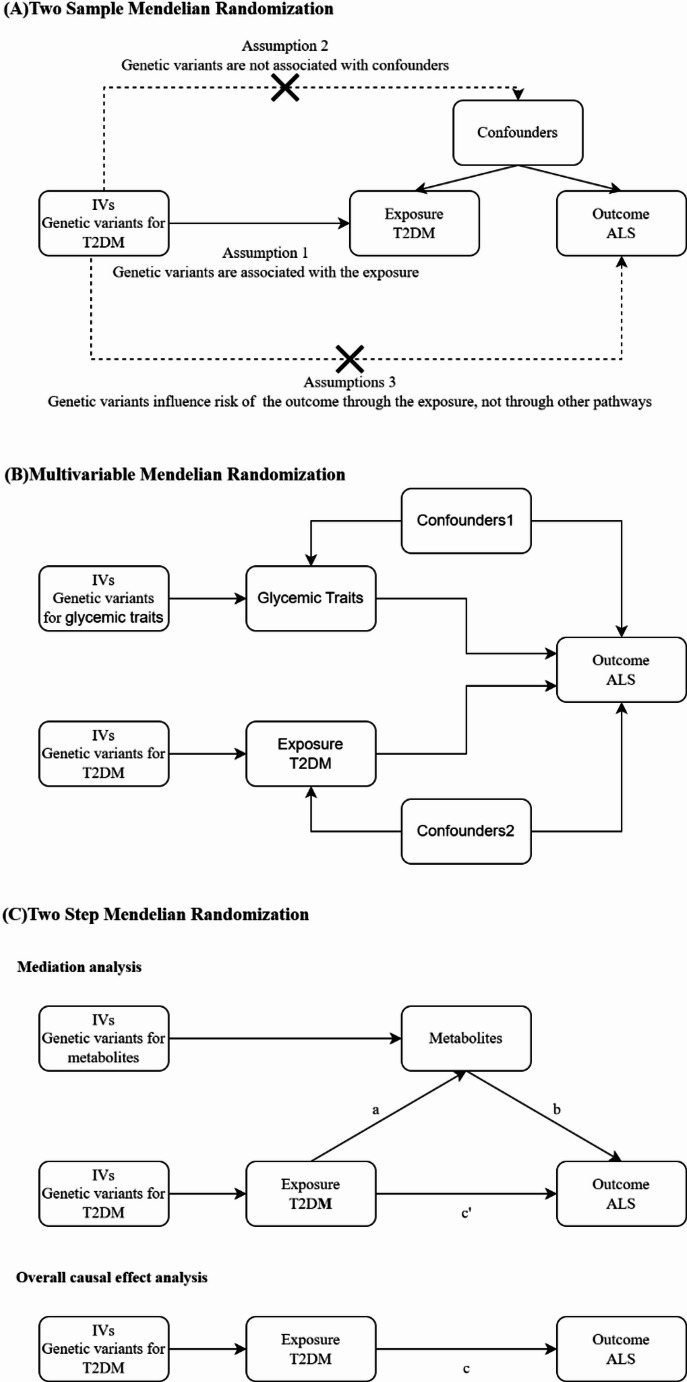
Overview of the study design. (**A**) The framework of the two-sample mendelian randomization. **B** The framework of the multivariable mendelian randomization (**C**) The framework of the two-step mendelian randomization. IVs, instrumental variables; T2DM, Type 2 Diabetes Mellitus; ALS, Amyotrophic Lateral Sclerosis

This study used a two-sample, two-step MR design (Fig. 1) to investigate the causal relationship between T2DM, blood metabolites, and ALS. The genetic variants serving as instrumental variables (IVs) met three key assumptions [11]: they were strongly associated with the exposure (relevance), independent of confounders (exchangeability), and affected the outcome only through the exposure (exclusion restriction). We followed the STROBE-MR guidelines [12] for transparent and rigorous reporting.

### Data sources

For T2DM, genetic associations were obtained from the FinnGen biobank (Risteys FinnGen R11-T2D) [13], a publicly available GWAS summary dataset. This approach was adopted to maintain the currency of the T2DM data and to minimize overlap with existing results or intermediate samples. Enhancing the sample diversity improves the generalizability of the findings. The dataset comprised 440,735 Finnish individuals, including 71,728 cases and 369,008 controls [13]. T2DM diagnoses were derived from hospital episode statistics, covering a range of conditions classified by ICD-10 codes, with type 1 diabetes cases excluded. The study utilized published GWAS results without accessing individual-level data.

Genetic associations for glycemic traits were sourced from the Meta-Analyses of Glucose and Insulin-related traits Consortium (MAGIC) [14]. The dataset included measures of fasting blood glucose (FG), two-hour postprandial blood glucose (2hGlu), fasting insulin (FI), and glycated hemoglobin (HbA1c). The study focused on European descent participants (*n* = 200,622) to minimize confounding due to ethnic genetic variation. Participants included those with diabetes diagnoses and individuals with elevated glucose levels, as defined by specific thresholds. Genetic associations were identified using multivariate linear regression, adjusted for age, sex, BMI (excluding HbA1c), study-specific covariates, and genomic control [14], ensuring sample representativeness and result validity.

Metabolomic marker genetic associations were obtained from UK Biobank summary statistics [15], involving 115,078 individuals. The metabolomic markers were assessed using an NMR spectroscopy platform from Nightingale Health, encompassing 168 biomarkers as detailed by Wong et al. [16]. Associations were evaluated using a linear mixed model, adjusted for population stratification, genetic relatedness, age, sex, fasting status, and genotyping array, to ensure robust statistical control and enhance finding reliability.

For ALS, genetic associations were derived from the large-scale, European-based ALS GWAS summary data of Project MinE [17]. The dataset included 138,086 participants of European ancestry, with 27,205 ALS patients and 110,881 controls. ALS diagnoses were made according to the revised El Escorial criteria [18] by specialists in motor neuron disease. All participants were recruited from specialized ALS clinics, ensuring high diagnostic accuracy, with controls having no signs of ALS or other neurodegenerative diseases [18].

### Selection of IVs and data harmonization

To ensure robust causal inference, we carefully selected IVs and harmonized the data. We screened for single nucleotide variants (SNVs) with a strong association with the exposure using a significance threshold of *p* < 5 × 10^−8^. To eliminate linkage disequilibrium (LD) and ensure SNV independence, we applied LD clumping with an r^2^ threshold of 0.001 and a window size of 10,000 kb [19]. Palindromic SNVs with intermediate allele frequencies were excluded, and only SNVs with a minor allele frequency (MAF) > 1% were retained. To avoid weak instrument bias, we excluded SNVs with F statistics below 10 [20]. Additionally, to prevent reverse causation, we used the Steiger filter to confirm the directionality of each SNV based on its explanatory power for the exposure [21]. SNVs with incorrect Steiger direction or a Steiger *p* < 0.05 were excluded. We aligned the effect estimates of the instrumental SNVs for each exposure with those of the SNVs for the outcomes. For instrumental variants lacking matching SNVs in the outcome summary statistics, we identified proxies in LD with an r^2^ > 0.8. These quality control measures minimized biases due to LD or weak instruments, enhancing the validity and accuracy of our causal inference.

### MR analysis

#### Effect of T2DM on ALS by two-sample Mendelian randomization (TSMR)

To investigate the causal relationship between T2DM and ALS, we conducted a TSMR analysis. Using IVW regression under a random-effects model, we estimated the overall causal effect of T2DM on ALS. This method aggregates individual Single Nucleotide Variant based Wald ratio estimates to derive a comprehensive causal effect estimate by dividing the exposure effect by the outcome effect for each SNV using a random-effects model [22]. To enhance robustness and address potential horizontal pleiotropy, we supplemented IVW with MR-Egger, weighted-median, weighted-mode, and simple mode analyses. These methods collectively provide a comprehensive evaluation of the T2DM-ALS link. MR-Egger assesses the consistency of causal inferences across multiple SNVs while accounting for pleiotropy [23]; weighted-median estimation remains reliable even if up to half of the instruments are invalid; and mode analyses identify consistent causal effects among clusters of SNVs pointing in the same direction [24]. For exposures represented by a single SNV, we applied the Wald ratio test as a direct method to estimate the causal effect, treating the SNV as an instrumental variable. These complementary approaches offer a thorough assessment of the T2DM-ALS relationship, addressing potential pleiotropic effects.

#### Multivariate Mendelian randomization (MVMR) analysis

Building on our previous finding of a significant causal association between T2DM and ALS [25], we conducted a MVMR to explore the underlying mechanisms. MVMR controls for potential horizontal pleiotropy by including multiple exposures in the same model, providing more accurate results [26]. To isolate the independent effect of T2DM on ALS, we adjusted for glycemic traits and body mass index using a multivariate IVW approach, ruling out confounding effects from overlapping instrumental variables or other correlated factors [27].

#### Mediation analysis with metabolomic markers

We further investigated the mediating role of blood metabolites in the T2DM-ALS relationship using a two-step MR design. Initially, we assessed the impact of T2DM on blood metabolites via two-sample MR (Fig. 1C, Part a). Subsequently, we applied MVMR to evaluate the effects of metabolites significantly associated with T2DM on ALS risk (Fig. 1C, Part b). The total effect of T2DM on ALS (c) was decomposed into direct (c’) and indirect (a×b) components, quantified using the Sobel test. By dividing the indirect effect by the total effect, we calculated the proportion mediated by each metabolite. Confidence intervals were computed using the delta method, allowing us to examine the complex interplay between variables and determine the influence of mediating factors.

#### Statistical and sensitivity analyses

Statistical significance was set at *p* < 0.05, with Bonferroni corrections for multiple testing [28]. All analyses were conducted using R (version 4.4.0) with the “TwoSampleMR”, “MendelianRandomization”, and “MVMR” packages. To prevent reverse causality, we excluded SNVs using the Steiger filter [21]. Heterogeneity among SNVs was evaluated using funnel plots and Cochran’s Q test, with smaller p-values indicating greater heterogeneity and potential directional pleiotropy [29]. The MR-Egger intercept method was employed to detect horizontal pleiotropy, with a significant intercept (*p* < 0.05) suggesting its presence [30]. Leave-one-out analysis confirmed the stability of the causal estimates, ensuring that no single SNV disproportionately influenced the results [31].

## Result

### Basic information and instrumental variable selection

A summary of the contributing GWAS is presented in sTable 1. We identified instrumental variables for various traits: 36 SNVs for T2DM, 60 for 2hGlu, 56 for FI, 23 for FG, 48 for HbA1c, and 49 for metabolites. The average F statistics were 68.644 for T2DM, 56.362 for 2hGlu, 51.801 for FI, 133.615 for FG, 98.386 for HbA1c, and 143.655 for metabolites, with each SNV exceeding an F statistic of 10, suggesting robustness against weak instrument bias. Further details on these instrumental variables are available in sTables 2–7.

TSMR.

**Fig. 2:**
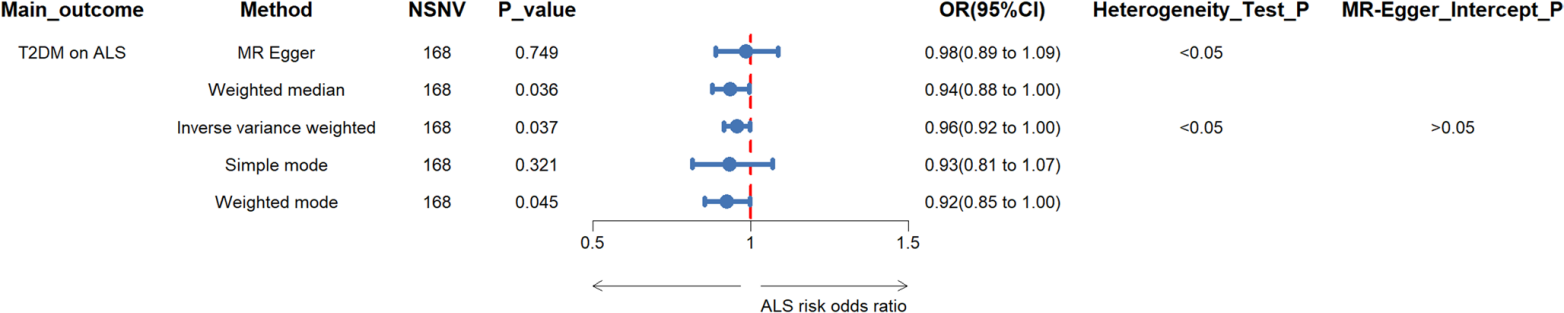
Forest Plot Showing the Associations Between the T2DM and ALS Risk. NSNV, number of independent genome-wide significant single nucleotide variations; OR = odds ratio; CI = confidence interval; T2DM, Type 2 Diabetes Mellitus; ALS, Amyotrophic Lateral Sclerosis

Employing the IVW method under a random-effects model, we estimated the causal effect of T2DM on ALS. The Steiger filter revealed no SNVs indicative of reverse causality. Cochran’s Q test and MR-Egger regression (Cochran’s Q = 254.217; *p* < 0.050) indicated heterogeneity among the instrumental variables, which was accounted for by the random-effects model. The MR-Egger intercept was not significantly different from zero (Intercept = −0.002; *p* > 0.050) (Fig. 2), ruling out significant horizontal pleiotropy. The odds ratio (OR) for ALS per 1 standard deviation (SD) increase in T2DM was 0.956 (95% CI 0.916–0.997; *p* = 0.037) (Fig. 2), suggesting a decreased risk of ALS with higher T2DM predisposition. This relationship was consistently supported by other MR methods including MR-Egger, weighted-median, and mode analyses (Fig. 2). Scatter and funnel plots (sFigs. 1 and 2) visualized the T2DM-ALS association, and leave-one-out analysis (sFigure 3) confirmed the robustness of our findings. These results are in line with previous studies highlighting a neuroprotective role of T2DM in ALS development [10, 25, 32].

### MVMR

Our previous study found no significant correlation between blood glucose characteristics and ALS risk [25]. To investigate whether T2DM’s protective effect on ALS is mediated through glycemic traits, we conducted MVMR, adjusting for four primary glycemic traits. After adjustment, T2DM maintained a significant causal association with ALS (OR = 0.928; 95% CI 0.886–0.973; *p* = 0.002), aligning with TSMR results. MR-Egger and multivariable median methods supported the IVW findings, offering reliable estimates across different scenarios (Table 1). This suggests that T2DM’s protective effect on ALS is not mediated through glycemic traits.

**Table 1:** Mendelian randomization analyses of the causal effects between T2DM and ALS after correction for glycemic traits

Exposure	Confounders	Method	NSNVs	β	SE	OR (95% CI)	*P* Value
T2DM	Glycemic Traits	Multivariable MR-Egger	202	−0.074	0.024	0.928 (0.886–0.973)	0.002
		Multivariable IVW	202	−0.075	0.024	0.928 (0.885–0.973)	0.002
		Multivariable median	202	−0.073	0.029	0.930 (0.879–0.983)	0.011
Abbreviations: *T2DM* Type 2 Diabetes Mellitus, ALS Amyotrophic lateral sclerosis, *NSNVs* number of independent genome-wide significant single nucleotide variations, *SE *Standard Error, *OR *odds ratio, *CI* confidence interval, *IVW* inverse-variance weighted							

### Two-step MR analysis

**Fig. 3:**
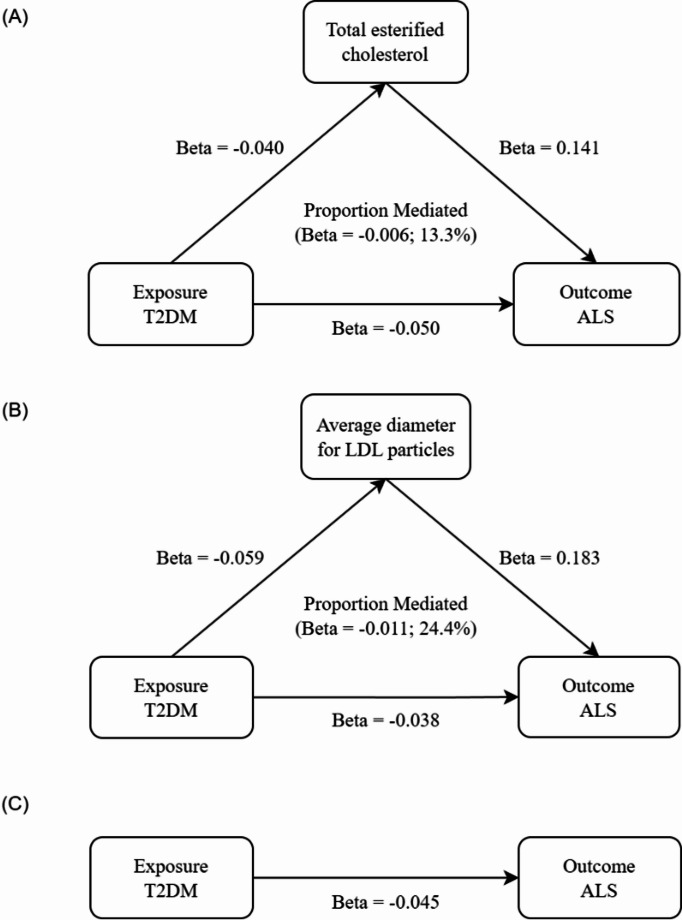
The potential causal evidence summarized from the MR analysis. T2DM, Type 2 Diabetes Mellitus; ALS Amyotrophic Lateral Sclerosis

We evaluated the effect of T2DM on 168 circulating metabolites and identified 704 instances of SNVs involving reverse causality using the Steiger filter, and excluded them from further analysis (sTable 8). 95 metabolites exhibited a significant association with T2DM [Bonferroni-corrected *p* < 2.98 × 10^−4^ (0.050/168)] (sTable 9, and 10). After removing one result indicative of pleiotropy (sTable 11), we examined the effect of these 94 metabolites significantly related to T2DM on the risk of ALS (sTables 12, 13 and 14). No SNVs with reverse causality were detected by the Steiger filter. Our preliminary MR analysis identified five metabolites associated with ALS: triglycerides in large LDL particles (OR = 1.064; 95% CI 1.008–1.123; *p* = 0.026), cholesterol in very small very low-density lipoprotein (VLDL) particles (OR = 1.075; 95% CI 1.005–1.151; *p* = 0.036), average diameter of LDL particles (OR = 1.123; 95% CI 1.006–1.254; *p* = 0.038), cholesteryl esters in very small VLDL particles (OR = 1.078; 95% CI 1.006–1.155; *p* = 0.032), and TEC (OR = 1.104; 95% CI 1.012–1.203; *p* = 0.026). However, after adjusting for multiple testing using the Bonferroni correction (Bonferroni-corrected *p* > 5.32 × 10^−4^ [0.050/94]), none of these associations remained statistically significant. Subsequently, we analyzed four metabolites as potential mediators of the effect of T2DM on ALS using MVMR. Among the five initially identified metabolites, the direction of the mediating effect of triglycerides in large LDL particles was inconsistent with the overall effect, suggesting that this metabolite may not be a true mediator. Therefore, the remaining four metabolites—cholesterol in very small VLDL particles, average diameter of LDL particles, cholesteryl esters in very small VLDL particles, and TEC—were further analyzed to explore their roles in the protective effect of T2DM on ALS. Our findings indicated that the protective effect of T2DM on ALS is not attributable to alterations in blood glucose levels caused by T2DM, as MVMR analysis showed no significant association between T2DM and blood glucose levels (OR = 1.077; 95% CI 0.857–1.354; *p* = 0.525). In conjunction with results shown above, we systematically demonstrated that the protective effect of T2DM on ALS may not be mediated by glucose metabolism. Further investigation revealed that TEC levels accounted for 13.3% of the reduction in T2DM-associated ALS risk (proportion mediated = 13.3%; *p* < 0.050) (Table 2, sTable 15, Fig. 3). Additionally, the average diameter of LDL particles contributed 24.4% to the reduction in T2DM-associated ALS risk (proportion mediated = 24.4%; *p* < 0.050). These preliminary findings suggest that T2DM might contribute to a partial reduction in the risk of ALS by lowering the levels of TEC in the bloodstream. Additionally, T2DM may help mitigate the negative impact of average LDL particle diameter on the onset and progression of ALS.

**Table 2:** The mediation effect of T2DM on ALS via multiple metabolites

Mediator	Method	NSNVs	β	SE	OR (95% CI)	*P* Value
Cholesterol in very small VLDL	Multivariable MR-Egger	147	0.142	0.082	1.152(0.981–1.353)	0.084
	Multivariable IVW	147	0.145	0.078	1.156(0.992–1.346)	0.064
	Multivariable median	147	0.086	0.090	1.089(0.913–1.299)	0.342
Cholesteryl esters in very small VLDL	Multivariable MR-Egger	148	0.095	0.083	1.100(0.935–1.293)	0.251
	Multivariable IVW	148	0.115	0.079	1.122(0.961–1.309)	0.147
	Multivariable median	148	0.032	0.090	1.035(0.865–1.232)	0.722
Total esterified cholesterol	Multivariable MR-Egger	153	0.143	0.071	1.153(1.004–1.324)	0.043
	Multivariable IVW	153	0.141	0.069	1.152(1.006–1.319)	0.040
	Multivariable median	153	0.123	0.083	1.131(0.961–1.331)	0.140
Average diameter for LDL particles	Multivariable MR-Egger	146	0.180	0.084	1.197(1.015–1.412)	0.033
	Multivariable IVW	146	0.183	0.084	1.201(1.019–1.414)	0.029
	Multivariable median	146	−0.015	0.120	0.985(0.778–1.247)	0.901
Abbreviations: *T2DM* Type 2 Diabetes Mellitus, *ALS* Amyotrophic lateral sclerosis, *NSNVs *number of independent genome-wide significant single nucleotide variations, *SE* Standard Error, *OR* odds ratio, *IVW* inverse variance weighted						

## Discussion

Our study pioneers the application of MR analysis to explore the complex relationship between T2DM and ALS, with a focus on identifying potential intermediary roles of blood metabolites and alternative metabolic pathways. We employed strict inclusion criteria, sensitivity analyses, and multiple advanced MR techniques (TSMR, MVMR, and a two - step approach). Contrary to the prevailing hypothesis of a blood glucose - related mechanism [33], the results indicate that the protective effect of T2DM on ALS isn’t primarily mediated by glucose metabolism modulation. Instead, exploratory mediation analyses using MR techniques suggest two specific lipid metabolites (TEC and the average diameter of LDL particles) may be implicated in this pathway, potentially explaining up to 13.3% and 24.4%, respectively, of the observed association in this dataset. These findings position LDL particle diameter and TEC as hypothesis - generating markers worthy of further exploration, highlighting the possibility of protective mechanisms rooted in lipid composition and function rather than solely glucose homeostasis regulation.

**Fig. 4:**
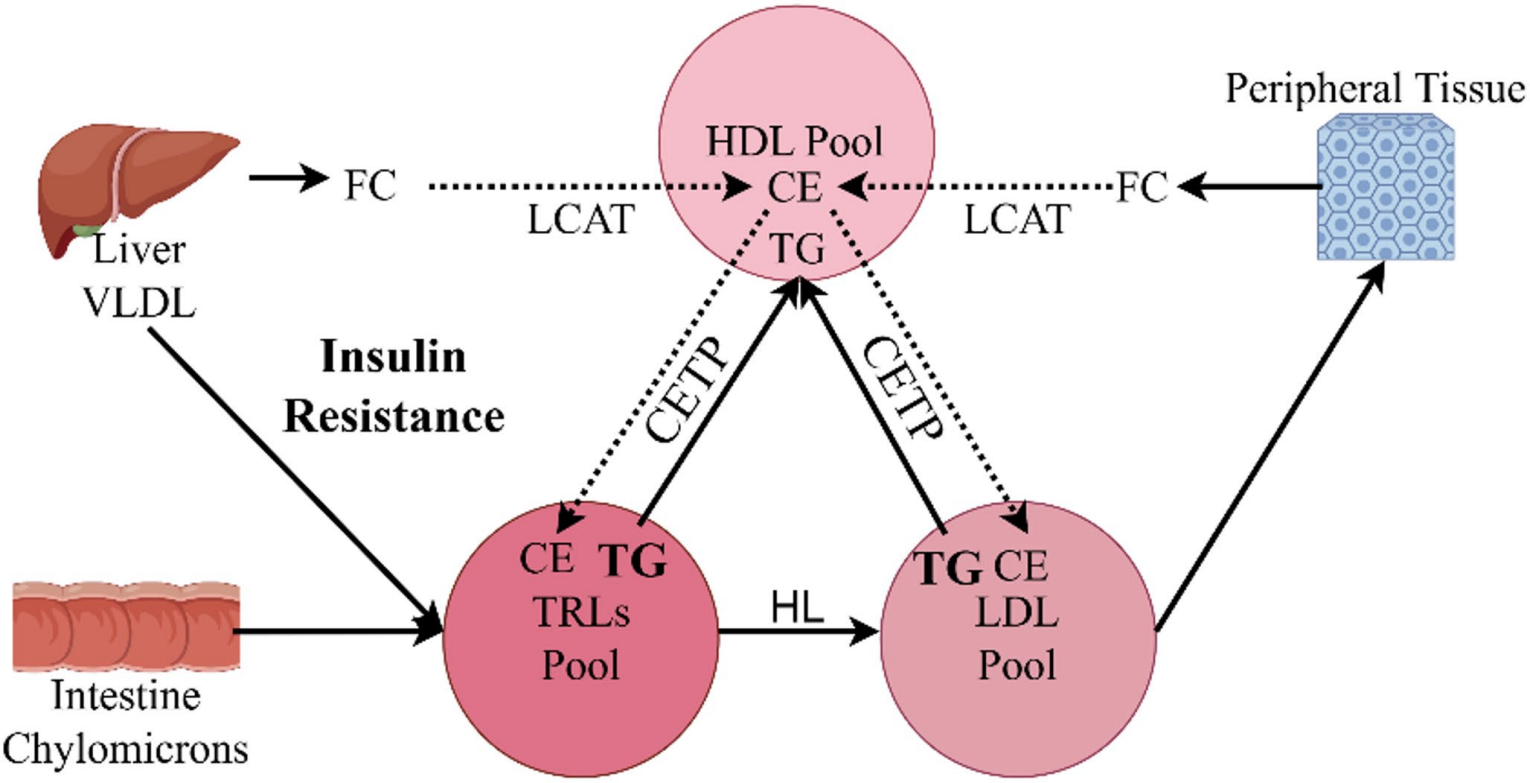
Disorder of lipid metabolism in type 2 diabetes mellitus. VLDL, very low-density lipoprotein; CE, cholesterol ester; FC, free cholesterol; TG triglyceride; TRLs, triglyceride rich lipoproteins; LDL, low density lipoprotein; HDL, high density lipoprotein; HL, hepatic lipase; LCAT, lecithin-cholesterolacyltransferase; CETP, cholesterolestertransferprotein. By Figdraw

This study builds on previous work [10, 25, 32, 34] by making several key contributions. First, we replicated the causal association between T2DM and reduced ALS risk in a European population. Second, using MVMR, we demonstrated that this protective effect is independent of glycemic traits. Third, our two - step MR analysis revealed the mediating role of TEC and LDL particle diameter on ALS risk, offering potential intervention targets. Furthermore, we explored the role of TEC in the relationship between T2DM and ALS. Our findings suggest that T2DM is associated with lower TEC levels, which may potentially protect against ALS [8]. TEC is crucial for cell membrane fluidity and integrity [35]. In ALS, disrupted cholesterol metabolism contributes to disease pathogenesis [36]. Esterified cholesterol, mainly transported by LDL, enters cells via LDL receptors and is hydrolyzed into free cholesterol, essential for cellular functions [37]. High LDL - C levels in ALS patients correlate with increased mortality, likely due to toxic lipid accumulation and oxidative stress [38–40]. In T2DM, reduced LCAT activity decreases TEC synthesis, consistent with our observations. LCAT transfers fatty acids from phosphatidylcholine to unesterified cholesterol on HDL and LDL [41]. Insulin resistance in T2DM promotes overproduction of triglyceride - rich VLDL particles and increases CETP activity [42, 43], leading to higher triglyceride content in LDL particles [44] and making them more susceptible to hepatic lipase degradation [45, 46] (Fig. 4). This altered LDL composition may contribute to the protective effects of T2DM against ALS.

Our study clarifies that changes in lipid metabolic pathways, especially those concerning esterified cholesterol homeostasis and LDL particle dynamics, might be crucial modulators of ALS pathogenesis. The association between T2DM and a lower ALS risk, potentially mediated by lipid - related mechanisms, underscores the key role of lipid metabolism homeostasis in ALS pathology. As a major product of liver cholesterol esterification, TEC’s level changes likely mirror the complex interplay between hepatic lipid transport and neuronal membrane stability [47]. The reduction in LDL particle diameter is linked to the atherogenic lipoprotein profile, implying that the distribution of lipoprotein subtypes may be involved in the disease process by affecting neuroinflammation or oxidative stress [48]. This suggests that dysregulation of hepatic lipid metabolism or interactions from gut microbiota - derived metabolism might influence disease susceptibility. Although the protective effect of T2DM seems independent of glycaemic control, lipidomic parameters like TEC and LDL particle diameter warrant further research into the two - way link between metabolic syndrome and neurodegeneration. These results highlight the need for longitudinal studies to determine whether lipid metabolites can serve as early biomarkers for assessing ALS risk. They also stress the importance of understanding the molecular networks connecting lipid metabolism with motor neuron degeneration. Future research should investigate how hepatic lipid synthesis pathways, intestinal lipid absorption mechanisms, and microbial bile acid metabolism contribute to this complex interaction, potentially revealing new insights into the causes and development of ALS.

## Conclusion

This study thoroughly investigates the causal relationships among T2DM, plasma metabolites, and ALS. Our exploratory mediation analysis using MR methods indicates that T2DM’s protective effect on ALS may not be mediated by glucose metabolism, but rather through TEC and the average diameter of LDL particles. These findings highlight the metabolic interplay between T2DM and ALS, laying a foundation for future research to validate these associations and explore their mechanisms. This may help identify high - risk individuals and inform targeted nutritional interventions to reduce ALS risk.

## Electronic Supplementary Material

Below is the link to the electronic supplementary material.

Supplementary Material 1 (XLSX 1.54 MB)

Supplementary Material 2 (DOCX 357 KB)

Supplementary Material 3 (DOCX 18.7 KB)

## Data Availability

The original contributions of this study are detailed within the article and supplementary materials. For additional inquiries, please contact the corresponding author.
