# Supplementary material for: Two-step Mendelian randomization reveals a lipid-driven protective effect of type 2 diabetes on ALS
Source: Neurol Sci. 2025 Aug 18;46(10):5133–44. doi: 10.1007/s10072-025-08407-0 (PMC12488786; doi:10.1007/s10072-025-08407-0)
Supplement: Supplementary file 2 — Supplementary Material 2 (DOCX 357 KB) [file 10072_2025_8407_MOESM2_ESM.docx]

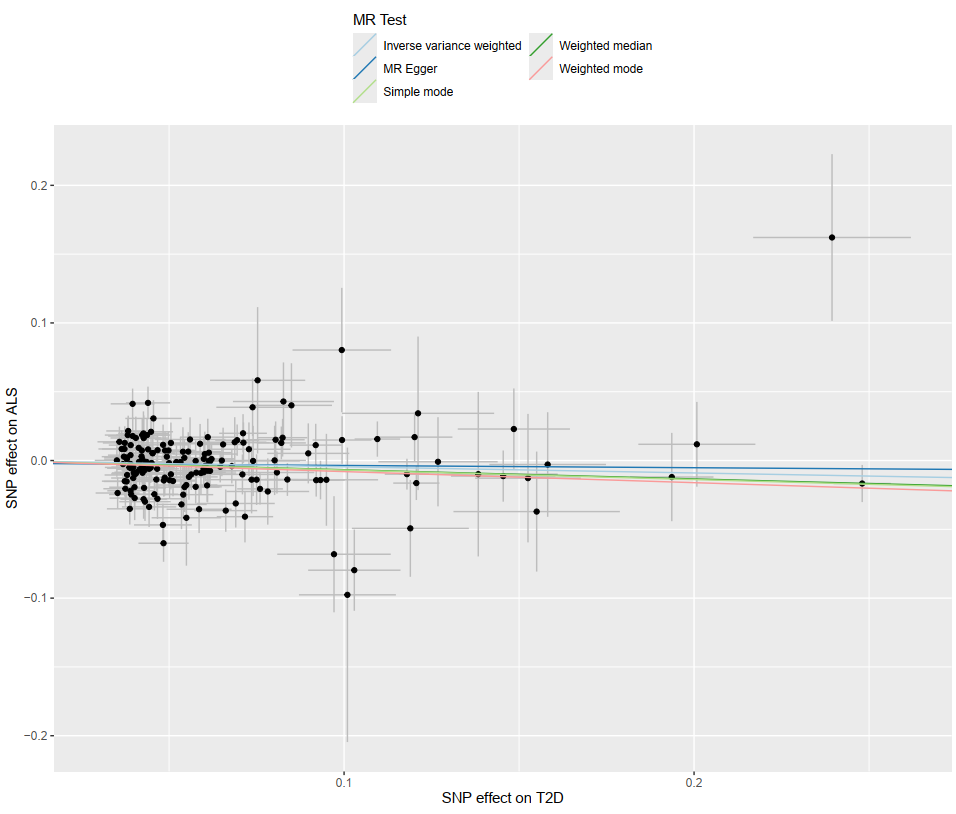


sFigure 1: Scatter plots for MR analyses showing the causal estimates of T2DM on ALS risk. The effect sizes of each SNV with 95% confidence intervals are represented by black points and grey error bars. The slope of each line shows the estimated MR effect for each method. T2DM, type 2 diabetes mellitus; ALS, Amyotrophic lateral sclerosis.


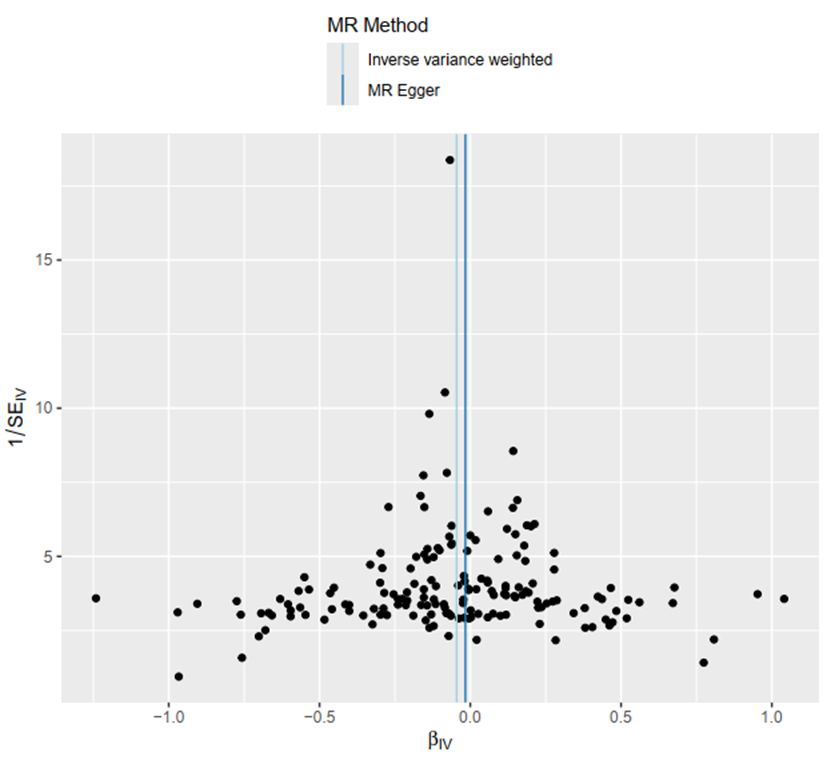


sFigure 2: Mendelian randomization funnel plot of T2DM and ALS association.


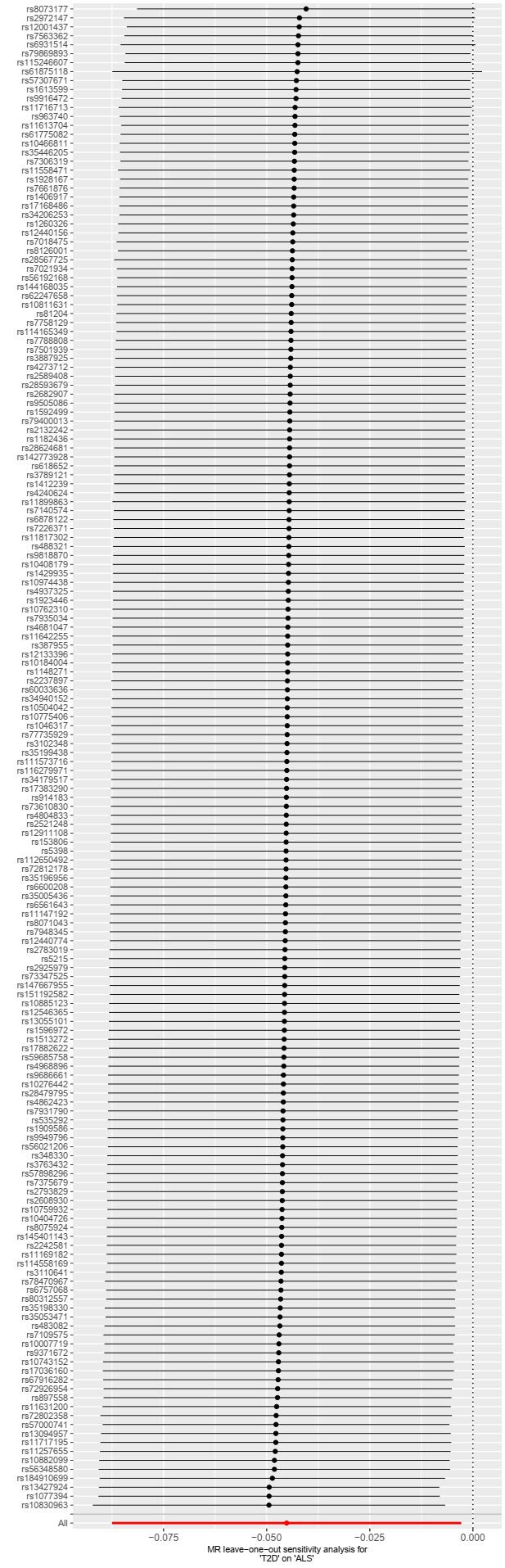


sFigure 3: Leave-one-out analysis for T2DM on ALS
