# Supplementary material for: Two-step Mendelian randomization reveals a lipid-driven protective effect of type 2 diabetes on ALS
Source: Neurol Sci. 2025 Aug 18;46(10):5133–44. doi: 10.1007/s10072-025-08407-0 (PMC12488786; doi:10.1007/s10072-025-08407-0)
Supplement: Supplementary file 3 — Supplementary Material 3 (DOCX 18.7 KB) [file 10072_2025_8407_MOESM3_ESM.docx]

| sTable 14 The mediation effect of T2DM on ALS via glucose metabolism. | | | | | | | |
| --- | --- | --- | --- | --- | --- | --- | --- |
| Type | Method | No.SNV | β | SE | OR (95% CI) | | P Value |
| Exposure |  |  |  |  |  |  |  |
| T2DM | Multivariable IVW | 140 | -0.065 | 0.031 | 0.937(0.882-0.996) | | 0.036 |
| Mediator |  |  |  |  |  |  |  |
| Metabolite-Glucose | Multivariable MR-Egger | 140 | 0.072 | 0.117 | 1.075 (0.855-1.352) | | 0.536 |
|  | Multivariable IVW | 140 | 0.074 | 0.117 | 1.077 (0.857-1.354) | | 0.525 |
|  | Multivariable median | 140 | -0.028 | 0.135 | 0.972 (0.745-1.268) | | 0.834 |
| Abbreviations: T2DM = Type 2 Diabetes Mellitus; ALS = Amyotrophic lateral sclerosis; No.SNV = number of independent genome-wide significant single nucleotide variations; SE = Standard Error; OR = odds ratio; CI = confidence interval; IVW = inverse-variance weighted. | | | | | | | |

| sTable 15 The Mediating Role of Metabolites in the Effect of T2DM on ALS. | | | | |
| --- | --- | --- | --- | --- |
| Mediated Pathway  (T2DM to ALS) | Indirect Effect  (95% CI) | Total Effect  (95% CI) | Proportion Mediated  (%) | P Value |
| Via Cholesterol in very small VLDL | -0.005(-0.011, 0) | -0.045(-0.087, -0.003) | 11.1 | 0.096 |
| Via Cholesteryl esters in very small VLDL | -0.005(-0.012, 0.002) | -0.045(-0.087, -0.003) | 11.1 | 0.096 |
| Via Average diameter for LDL particles | -0.011(-0.022, -0.001) | -0.045(-0.087, -0.003) | 24.4 | 0.028 |
| Via Total esterified cholesterol | -0.006(-0.012, 0) | -0.045(-0.087, -0.003) | 13.3 | 0.046 |
| Abbreviations: T2DM = Type 2 Diabetes Mellitus; ALS = Amyotrophic lateral sclerosis; NSNV = number of independent genome-wide significant single nucleotide variations; SE = Standard Error; CI = confidence interval; VLDL, very low-density lipoprotein; LDL, low-density lipoprotein | | | | |
